# Ceramide Metabolism Regulated by Sphingomyelin Synthase 2 Is Associated with Acquisition of Chemoresistance via Exosomes in Human Leukemia Cells

**DOI:** 10.3390/ijms231810648

**Published:** 2022-09-13

**Authors:** Makoto Taniguchi, Shingo Nagaya, Kohei Yuyama, Ai Kotani, Yasuyuki Igarashi, Toshiro Okazaki

**Affiliations:** 1 Medical Research Institute, Kanazawa Medical University, Uchinada 920-0293, Japan; 2 Research Institute for Bioresources and Biotechnology, Ishikawa Prefectural University, Nonoichi 921-8836, Japan; 3 Material Research Department, Shalom Co., Ltd., Oshino 401-0511, Japan; 4 Lipid Biofunction Section, Frontier Research Center for Advanced Material and Life Science, Faculty of Advanced Life Science, Hokkaido University, Sapporo 001-0021, Japan; 5 Department of Hematological Malignancy, Institute of Medical Sciences, Tokai University, Isehara 259-1193, Japan

**Keywords:** ceramide, doxorubicin, drug resistance, exosome, leukemia, microRNA, *miR-484*, sphingomyelin, sphingomyelin synthase

## Abstract

Ceramide levels controlled by the sphingomyelin (SM) cycle have essential roles in cancer cell fate through the regulation of cell proliferation, death, metastasis, and drug resistance. Recent studies suggest that exosomes confer cancer malignancy. However, the relationship between ceramide metabolism and exosome-mediated cancer malignancy is unclear. In this study, we elucidated the role of ceramide metabolism via the SM cycle in exosomes and drug resistance in human leukemia HL-60 and adriamycin-resistant HL-60/ADR cells. HL-60/ADR cells showed significantly increased exosome production and release compared with parental chemosensitive HL-60 cells. In HL-60/ADR cells, increased SM synthase (SMS) activity reduced ceramide levels, although released exosomes exhibited a high ceramide ratio in both HL-60- and HL-60/ADR-derived exosomes. Overexpression of SMS2 but not SMS1 suppressed intracellular ceramide levels and accelerated exosome production and release in HL-60 cells. Notably, HL-60/ADR exosomes conferred cell proliferation and doxorubicin resistance properties to HL-60 cells. Finally, microRNA analysis in HL-60 and HL-60/ADR cells and exosomes showed that *miR-484* elevation in HL-60/ADR cells and exosomes was associated with exosome-mediated cell proliferation. This suggests that intracellular ceramide metabolism by SMS2 regulates exosome production and release, leading to acquisition of drug resistance and enhanced cell proliferation in leukemia cells.

## 1. Introduction

Ceramides are classified as sphingolipids and are bioactive lipids involved in cell death, inflammation, and autophagy as well as functioning as major components of the cellular membrane including the plasma membrane [1]. In particular, increased cellular ceramides in response to numerous cellular stimuli such as anti-cancer drugs induce various types of cell death including apoptosis, necroptosis, ferroptosis, and autophagy-regulated cell death [2]. Ceramides are generated from three pathways: de novo synthesis from L-serine and palmitoyl-CoA; the salvage pathway through recycling of other sphingolipids such as glucosylceramide (GC); and the ceramide/sphingomyelin (SM) cycle utilizing sphingomyelin hydrolysis by sphingomyelinase (SMase) [2,3]. Previously, we found that malignant drug-resistant leukemia cells maintained low levels of ceramides via enhancing SM synthesis by SM synthase (SMS) and GC synthesis by GC synthase (GCS) to prevent ceramide-mediated cell death induced by anti-cancer drugs [4]. In addition, ceramide generation from SM hydrolysis by SMase involves the suppression of cancer progression through induction of cell death in response to anti-cancer treatments such as agents or irradiation [2].

Exosomes are small double-membrane bound vesicles (30–150 nm in diameter) that are derived from endosomes and are released from cells [5]. Extracellular exosomes act as intercellular transporters of cellular cargo such as proteins, mRNA, microRNA, and lipids [5,6]. Many studies accumulated evidence that exosomes are involved in cancer malignancy by controlling cancer cell proliferation, cancer metastasis, and drug resistance via the delivery of their contents [7,8].

Recent research has revealed that exosomes are formed and released through multiple molecular mechanisms. One well-established pathway uses the endosomal sorting complex required for the transport (ESCRT)-dependent pathway. In the ESCRT pathway, four protein complexes (ESCRT-0, -I, II, and -III) are involved in exosome formation in early endosome and cargo sorting [5,9]. As in other pathways, membrane lipids and their metabolic enzymes have been reported to be associated with exosome formation and release [10,11]. In particular, ceramide biogenesis through the ceramide/SM cycle mediates the curvature and budding of vesicles [12]. In this pathway, suppression of ceramide generation by inhibition of neutral SMase (nSMase2) results in the decrease in exosome release. SMase-generated ceramides enhanced exosome release to transfer miRNAs [13,14]. Therefore, evidence is accumulating that the production and release of exosomes regulated by ceramide generation through the SM cycle is involved in various cellular physiological functions. Yuyama et al. demonstrated that regulation of neuron-derived exosome release by the SM cycle involves clearance of amyloid β-peptide [15]. In cancer, neutral SMase2-dependent exosomal transfer of miRNA enhances angiogenesis and cancer metastasis [16]. Inversely, treatment of extracellular C_6_-ceramide suppresses the proliferation and angiogenic activity of human multiple myeloma cells through the enhancement of exosome release containing tumor-suppressive miRNAs [17,18]. However, little is known about how intracellular ceramide production via the ceramide/SM cycle is associated with anti-cancer drug resistance and cancer malignancy through exosome generation and release.

Therefore, we examined this mechanism by utilizing drug-resistant human leukemia HL-60/ADR cells, which show resistance to anti-cancer drugs including adriamycin through an enhanced drug-efflux pump function, and their parental drug-sensitive HL-60 cells. In particular, the effect of intracellular ceramide metabolism by the ceramide/SM cycle on exosome production and release was investigated. In addition, we attempted to elucidate exosomal functions related to the drug resistance and cancer cell proliferation by comparing the composition of microRNAs (miRNAs) in exosomes and cells.

## 2. Results

### 2.1. Exosome Release Is Promoted in Drug-Resistant Leukemia Cells

**Figure 1:**
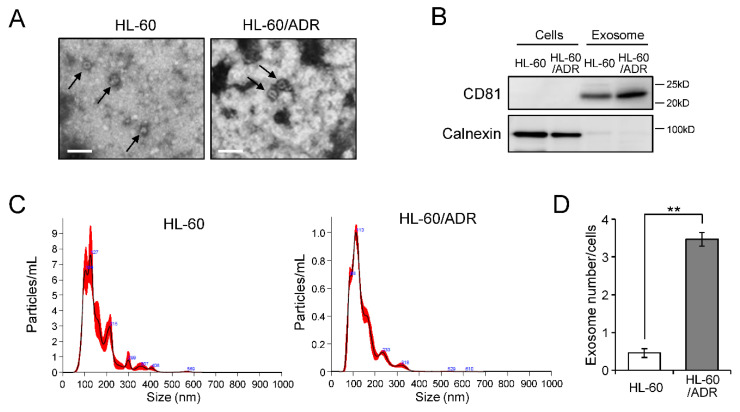
Enhancement of exosome release in HL-60/ADR cells. Exosomes were prepared from the culture media of HL-60 cells and HL-60/ADR cells by sequential ultracentrifugation methods. (**A**) Purified exosomes of HL-60 were observed by transmission electron microscopy. The arrow indicates exosome. Bars, 250 nm. (**B**) Western blot analysis of exosome marker CD81 and endoplasmic reticulum marker calnexin in exosomes and cells. (**C**,**D**) Exosome size and number were analyzed by nanosight tracking analysis. Particle size distribution of exosomes purified from HL-60 (left) and HL-60/ADR cells (right) are represented (**C**). Exosome numbers were normalized by cell numbers (**D**). Values indicate mean ± SD (*n* = 3). ** *p* < 0.005.

To examine the relationship between exosome production and the drug resistance of leukemia cells, we extracted exosomes from drug-sensitive HL-60 cells and adriamycin-resistant HL-60/ADR cells by ultracentrifugation. Exosomes extracted from both HL-60 and HL-60/ADR cells were detected as <200 nm particles under transmission electron microscopy (Figure 1A). Western blot analysis showed that exosomal marker CD81 was detected in exosomes without contamination of cellular organelles (Figure 1B and Figure S1). We then compared the size and number of HL-60 and HL-60/ADR exosomes by nanoparticle tracking analysis (Figure 1C,D). Although exosome size was the same between HL-60 cells and HL-60/ADR cells, at 100–200 nm, exosome numbers of HL-60/ADR cells were clearly higher than that of HL-60 cells (*p* = 0.0022) (Figure 1D). These results indicated that exosome release is more active in drug-resistant HL-60/ADR cells than in parental HL-60 cells.

### 2.2. Cellular Ceramide Levels Are Related to Exosome Release in HL-60/ADR Cells

**Figure 2:**
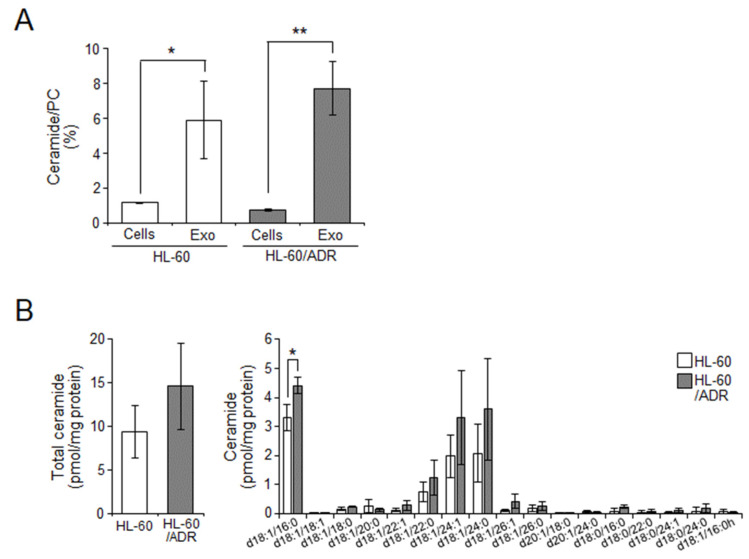
Ceramide contents in exosomes. Exosomal and cellular ceramides were measured by LC-MS/MS. (**A**) The ratio of ceramides was calculated by the amounts of phosphatidylcholine (PC). (**B**) Ceramide contents in exosomes. Values indicate mean ± SD (*n* = 3). * *p* < 0.05, ** *p* < 0.005.

Several reports have shown that cellular ceramides are related to exosome budding and their release from cells [12]. In addition, it has been reported that the amount and ratio of exosomal ceramides were high in metastatic colorectal cancer (CRC) cells or CRC patients compared with those in non-metastatic CRC cells or healthy cells [19]. Therefore, considering whether the ceramide ratio in exosomes affects exosome production and release, we next examined ceramide contents in exosomes and cells by liquid chromatography tandem mass spectrometry (LC-MS/MS). As expected, exosomes showed a higher ratio of ceramides than the cells of both lines (Figure 2A,B). However, the d18:1/d16:0 ceramide ratio was a little higher in HL-60/ADR exosomes than in HL-60 exosomes (Figure 2B). These data suggested that the ceramide contents of exosomes do not determine exosome release from cells in drug-resistant leukemia cells.

**Figure 3:**
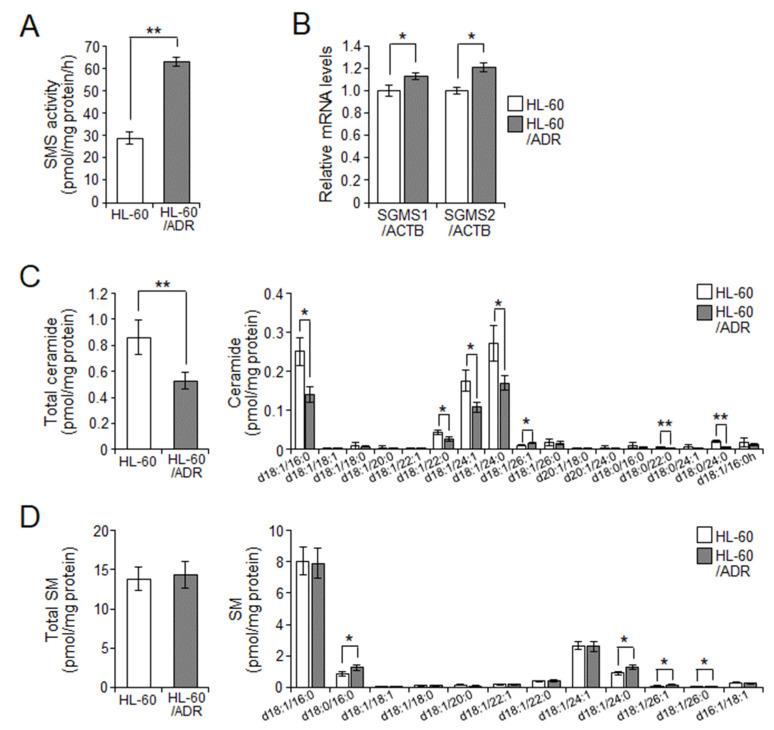
SMS regulates intracellular ceramide levels in HL-60/ADR cells. (**A**) Cellular SMS activity was analyzed using C_6_-NBD-ceramide as the substrate. (**B**) qPCR analysis of *SGMS1* (SMS1) and *SGMS2* (SMS2) in HL-60 and HL-60/ADR cells. Expression of SMSs was normalized against *ACTB* expression. (**C**,**D**) Sphingomyelin (SM) (**C**) and ceramide (**D**) levels were measured by LC-MS/MS. Values indicate mean ± SD (*n* = 3). * *p* < 0.05, ** *p* < 0.005.

It is known that SMS2, an SMS isoform, is involved in exosome production and its budding through SM and ceramide generation [15]. In addition, we previously showed that HL-60/ADR cells have higher SMS and GCS activity and lower ceramide levels compared with HL-60 cells (Figure 3A and Figure S2A,B) [4]. Therefore, we examined SM and ceramide production through SMS in HL-60 and HL-60/ADR cells (Figure 3). SMS has two isoforms, SMS1 and SMS2, which synthesize SM from ceramides and phosphatidylcholine [20]. As shown in Figure 3B, the expression of both SMS1 (*SGMS1*) and SMS2 (*SGMS2*) was higher in HL-60/ADR cells than in HL-60 cells. We next measured sphingolipid contents in HL-60 and HL-60/ADR cells by LC-MS/MS. Consistent with our previous data, ceramides, which are substrates of SMS, were decreased in HL-60/ADR cells compared with those in HL-60 cells (Figure 3C). However, total SM contents in HL-60/ADR cells were the same as that in HL-60 cells, even though some species of SM including d18:0/16:0, d18:1/24:0, d18:1/26:1, and d18:1/26:0 were higher in HL-60/ADR cells than in HL-60 cells (Figure 3D). In addition, GC levels were also unchanged between HL-60 and HL-60/ADR cells, although GCS activity in HL-60/ADR cells was higher than that in HL-60 cells (Figure S2A,B). These data suggested that suppression of intracellular ceramide levels through enhancement of SMS activity may be related to the elevation of exosome production and release in HL-60/ADR cells.

### 2.3. SMS2 Contributes to Exosome Release by Suppression of Ceramide Levels

**Figure 4:**
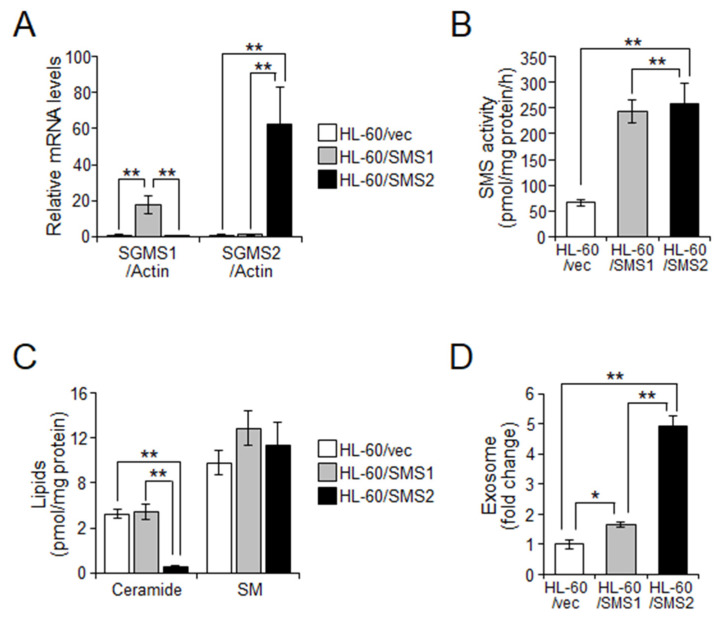
Overexpression of SMS2 increases exosome release in HL-60 cells. HL-60 cells overexpressing SMS1 or SMS2 were established by retroviral infection of hSMS1 (HL-60/SMS1) and hSMS2 (HL-60/SMS2). As a control, retrovirus vector containing empty vector was used (HL-60/vec). (**A**) qPCR analysis of *SGMS1* (SMS1) and *SGMS2* (SMS2) in HL-60/vec, HL-60/SMS1, and HL-60/SMS2 cells. Expression was normalized against *ACTB* expression (*n* = 3). (**B**) Cellular SMS activity was analyzed using C_6_-NBD-ceramide as the substrate (*n* = 3). (**C**) Ceramide and SM contents were measured by LC-MS/MS and normalized against cellular protein amounts (*n* = 4). (**D**) Exosome numbers were measured by Nanosight (*n* = 3). Values indicate mean ± SD. * *p* < 0.05, ** *p* < 0.005.

The above data demonstrated the possibility that intracellular ceramides enhanced exosome release in drug-resistant cells. Therefore, to examine whether the suppression of ceramide levels by increased SMS activity affects exosome release, we established HL-60 cells expressing SMS1 or SMS2 (Figure 4A). In HL-60 cells, both SMS1 and SMS2 similarly increased SMS activity (Figure 4B). However, ceramide levels were decreased only when SMS2 was overexpressed but not SMS1 (Figure 4C). SM levels were unchanged when both SMS1 and SMS2 were overexpressed (Figure 4C). Next, we elucidated exosome release in SMS-overexpressing cells. As shown in Figure 4D, HL-60/SMS2 cells showed a 4.951 ± 0.342-fold increase in the number of released exosomes compared with HL-60/vec cells and a 2.992 ± 0.207-fold increase over HL-60/SMS1 cells. However, SMS1 overexpression also slightly increased exosome release (1.654 ± 0.088-fold). These data suggested that SMS2 mainly regulates exosome release from cells through suppression of ceramide levels.

### 2.4. Effect of Exosomes Derived from HL-60/ADR Cells on Cell Proliferation and Drug Resistance

**Figure 5:**
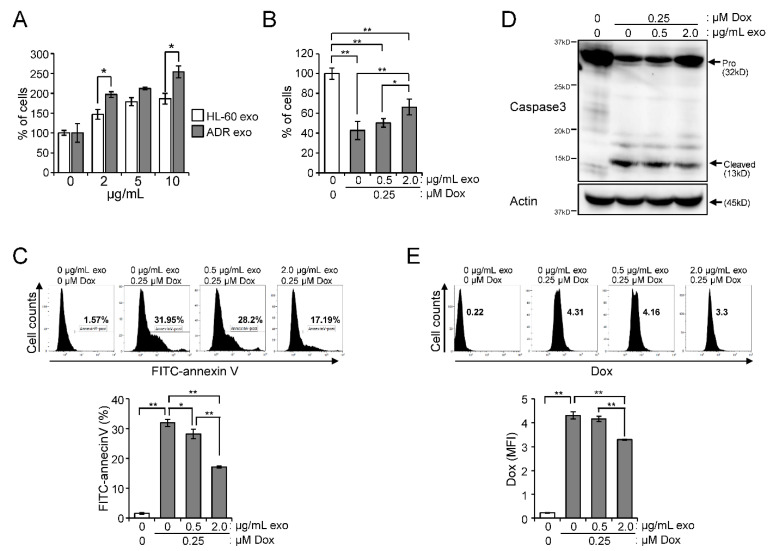
Exosomes purified from HL-60/ADR suppress doxorubicin-induced apoptosis in HL-60 cells. (**A**) HL-60 and HL-60/ADR cells were treated with exosomes extracted from the media of HL-60 (HL-60 exo) or HL-60/ADR cells (ADR exo) at the indicated concentration and cultured in RPMI-1640 supplemented with 5 μg/mL insulin and 5 μg/mL transferrin. After 12 h, viable cells were measured by CCK8 assay and represented as percentage of vehicle treatment (0 μg/μL) (*n* = 3). (**B**–**E**) HL-60 cells were pre-treated with ADR exo for 12 h and then treated with doxorubicin (Dox) for 12 h. (**B**) Viable cell numbers were counted after trypan blue exclusion and indicated as percentage of vehicle treatment (*n* = 4). (**C**) Cells were stained with FITC-conjugated annexin V and analyzed by flow cytometry (*n* = 3). (**D**) Western blot analysis of caspase-3 and actin. (**E**) Dox taken up by cells was analyzed by flow cytometry (*n* = 3). Values indicate mean ± SD. * *p* < 0.05, ** *p* < 0.005.

To investigate how exosomes of HL-60/ADR are associated with cancer cell proliferation and anti-cancer drug resistance, we treated HL-60 cells with exosomes extracted from HL-60 or HL-60/ADR cell culture media. Although treatment with both HL-60 and HL-60/ADR exosomes induced the proliferation of HL-60 cells in serum-free conditions, the effect was higher with HL-60/ADR exosomes than with HL-60 exosomes (Figure 5A). Doxorubicin (Dox), which is major anti-cancer drug against leukemia, induced apoptosis in HL-60 cells, while HL-60/ADR cells had resistance against Dox (Figure 5B,C and Figure S3). As expected, HL-60 exosome treatment did not confer the resistance to Dox on HL-60 ells (Figure S4). In contrast, treatment with HL-60/ADR exosomes decreased cell death induced by Dox in HL-60 cells (Figure 5B,C). In addition, HL-60/ADR exosomes suppressed caspase-3 activation induced by Dox in HL-60 cells (Figure 5D). Next, to examine whether drug elimination is enhanced by HL-60/ADR exosome treatment in HL-60 cells, the captured Dox was detected by flow cytometry. The remaining intracellular Dox was higher in HL-60 cells than in HL-60/ADR cells (Figure 5E and Figure S3D). Intracellular Dox fluorescence was decreased in HL-60 cells treated with HL-60/ADR exosomes (Figure 5E). These data suggested that HL-60/ADR exosomes confer drug-resistant properties to drug-sensitive HL-60 cells by enhancing the Dox elimination.

### 2.5. miR-484 Is Associated with HL-60 Cell Proliferation through Exosomes

**Figure 6:**
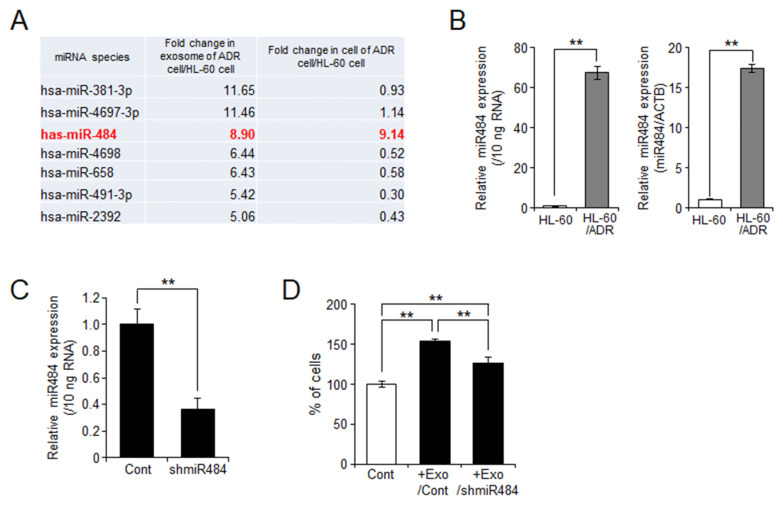
Exosomes purified from HL-60/ADR suppress doxorubicin-induced apoptosis in HL-60 cells. (**A**) Microarray experiments were performed to identify miRNAs in exosomes and cells. The seven candidate miRNAs showed more than a 5.0-fold increase among the total 2563 miRNAs species in HL-60/ADR cells compared with those in HL-60 cells. (**B**) Validation of *miR-484* levels in exosomes (left) and cells (right) was performed by qPCR. (**C**) Knockdown of *miR-484* by shRNA (shmiR-484) was performed by transfection of pLKO.1-shmiR-484 vector in HEK293 cells. Control cells were introduced with pLKO.1 control vector. The expression of *miR-484* in exosomes was analyzed by qPCR. (**D**) HL-60 cells were treated with 3 × 10^9^ exosomes from HEK293/control (Exo/Cont) or HEK293/shmiR-484 (Exo/shmiR-484) for 24 h. Viable cells were detected by trypan blue dye exclusion method. The values show the mean ± SD (*n* = 3). ** *p* < 0.005.

The above data showed that HL-60/ADR exosomes induced cell proliferation more strongly than HL-60 exosomes (Figure 5A). miRNAs were recently demonstrated to be involved in cancer cell proliferation [21]. To elucidate the differences between HL-60 and HL-60/ADR exosomes, we compared exosomal and cellular miRNA expression in HL-60 and HL-60/ADR cells by miRNA microarray. In the exosomes of HL-60/ADR cells, we identified seven miRNA candidates (*miR-381-3p*, *miR-4697-3p*, *miR-484*, *miR-4698*, *miR-658*, *miR491-3p*, and *miR2392*) with more than five-fold higher expression than in HL-60 cells (Figure 6A). Among them, *miR-484* only showed higher expression in both exosomes and cells. qPCR analysis confirmed elevation of *miR-484* expression in HL-60/ADR exosomes and cells compared with that in HL-60 cells (Figure 6B). To analyze the function of *miR-484* in cell proliferation in more detail, we performed *miR-484* knockdown in human embryonic kidney fibroblast HEK293 cells, which express *miR-484*. *miR-484* expression in the exosomes was decreased in *miR-484*-knockdown HEK293 cells compared with that in the control cells (Figure 6C). Finally, we examined the effects of exosomal *miR-484* levels on HL-60 cell proliferation. As shown in Figure 6D, control exosomes extracted from HEK293/control cells (Exo/Cont) increased the proliferation of HL-60 cells. However, *miR-484*-knockdown exosomes (Exo/shmiR-484) had a lower effect on HL-60 cell proliferation (Figure 6D). These results suggested that *miR-484*, which is highly expressed in HL-60/ADR cells compared with that in HL-60 cells, may activate cell proliferation via exosomes.

## 3. Discussion

Exosomes are a well-known transporter of cellular molecules including proteins, nucleotides, and lipids [5]. Recent studies have shown that exosome-mediated cell-to-cell interactions are involved in cancer cell proliferation, metastasis, and drug resistance, leading to cancer malignancy [8]. In this study, we found that exosome production and release are enhanced in adriamycin-resistant leukemia HL-60/ADR cells compared with those in parental HL-60 cells, and SMS2 regulates intracellular ceramide metabolism, resulting in the acquisition of drug resistance and increased proliferation. In drug-resistant HL-60/ADR cells, intracellular ceramides were maintained at low levels through increased SMS activity. Therefore, overexpression of SMSs in HL-60 cells increased exosome production and release, suggesting that intracellular ceramide/SM levels are critical regulators of this process. In addition, exosomes derived from HL-60/ADR conferred drug-sensitive HL-60 cells with cell proliferation and drug resistance properties. Finally, we identified *miR-484* as exosomal cargo that activates cancer cell proliferation.

Intracellular ceramide metabolism is an essential regulator of exosome production and release [12]. In addition, exosomal lipid compositions were reflected by cellular lipid conditions [10,11]. Previously, exosomal ceramides, especially C16:0 and C24:1 ceramides, were expressed at a higher ratio in metastatic CRC cells and CRC patient cells [19]. In addition, in glioblastoma, exosomal ceramide levels have been shown to be higher because they were closer to cancer stem cells [22]. These reports suggest an association between exosomal ceramides and cancer malignancy. Therefore, we first focused on ceramide levels in exosomes (Figure 2). However, the ceramide ratio in exosomes was almost the same between HL-60 cells and HL-60/ADR cells, and only C16:0 ceramide was slightly elevated in HL-60/ADR cells. Thus, we next examined the involvement between intracellular ceramide levels and exosomes that leads to chemoresistance.

Ceramide is well known as a bioactive lipid regulating cell death, differentiation, migration, and inflammatory responses [1,3]. In particular, in cancer, ceramide generation is known to be effective as a therapeutic target because it leads to cancer cell death and prevention of cancer malignancy [2]. Conversely, malignant cancer cells suppress ceramide production through enhancing its metabolism to SM or glycosphingolipids (GSLs) [23]. We previously reported that chemoresistant leukemia cells derived from patients have increased SMS and GCS activity, resulting in the maintenance of low ceramide levels [4]. In this study, HL-60/ADR cells also had higher SMS and GCS activity than HL-60 cells. In a recent study by Shammout et al., Dox-resistant human breast adenocarcinoma MCF-7 cells also showed lower ceramide levels and higher SMS2 expression compared with parental Dox-sensitive MCF-7 cells [24]. In particular, C16:0 and C22:0 ceramides in Dox-resistance MCF-7 cells were more than twice as high as in parental MCF-7 cells. Similarly, Huang et al. reported that C16:0 and C24:0 ceramides were decreased in taxol-resistant A2780 human ovarian cancer cells [25]. Inversely, SM levels became higher in chemoresistant cancer cells compared with levels in parental chemosensitive cells. In the present study, we confirmed increased SMS activity through the elevation of SMS expression including SMS1 and SMS2 and low ceramide levels including C16:0, C22:0, C24:1, and C24:0 ceramides in HL-60/ADR cells. However, total SM levels were similar between HL-60 cells and HL-60/ADR cells. Some species such as C16:0, C24:0, C26:0, and C26:1 SMs were slightly increased. Nonetheless, many studies have reported that the generation of GSLs including GC or hexosylceramide (HexCer) from ceramides is associated with chemoresistance [26]. Our previous study showed that overexpression of GCS enhanced resistance against Dox-induced cell death in HL-60 cells [4]. However, our data showed that total HexCer levels were not changed in HL-60/ADR cells compared with levels in HL-60 cells, although GCS activity was increased in HL-60/ADR cells (Figure S2). Therefore, these results suggest that suppression of ceramides is more important for chemoresistance in HL-60/ADR cells than increases in SM or GSLs from ceramides.

In our data, treatment of chemosensitive HL-60 cells with exosomes derived from chemoresistant HL-60/ADR cells enhanced cell proliferation and Dox-resistance (Figure 5). Exosomal cargos are associated with cancer malignancy including cell proliferation, metastasis, and chemoresistance [7,8]. In particular, miRNA is considered to be an important factor in exosome-mediated cancer malignancy [21]. Therefore, we finally examined the differences in exosomes between HL-60 cells and HL-60/ADR cells by miRNA microarray and identified seven miRNA candidates (*miR-381-3p*, *miR-4697-3p*, *miR-4698*, *miR-658*, *miR-491-3p*, *miR-2392*, and *miR-484*) with five-fold higher expression in HL-60/ADR exosomes than in HL-60 exosomes (Figure 6A). Among them, only *miR-484* was higher in both HL-60/ADR cells and exosomes than in HL-60 cells and exosomes. Therefore, we focused on the function of *miR-484* in cell proliferation. Recently, *miR-484* was reported to have both physiological and pathological functions [27]. In particular, *miR-484* expression exhibits the opposite role in cancer progression. Several reports showed that low *miR-484* expression increases the risk of cancer cell proliferation, migration, and invasion in gastric cancer [28,29], CRC [30,31,32], pancreatic cancer [33], and prostate cancer [34]. Conversely, overexpression of *miR-484* promotes tumorigenesis and malignancy in hepatocellular carcinoma [35,36,37], lung cancer [38,39,40], and glioma [41]. In our data, knockdown of *miR-484* suppressed exosome-mediated proliferation of HL-60 cells, suggesting that *miR-484* may be involved in cancer malignancy, at least in leukemia. However, the target of *miR-484* is unknown and requires further investigation. As for the other candidates, the increased expression of some of them is involved in cancer malignancy. Wu et al. showed that *miR-4697-3p* expression was significantly higher in carboplatin-resistant ovarian cancer OVCAR3 cells than in drug-sensitive MR182 cells [42]. *miR-4697-3p* increases chemoresistance through the transcriptional regulation of MNX1-AS1 and HOXB13. Moreover, *miR-4697-3p* expression is involved in the pathogenesis of gastric cancer [43]. *miR-658* was high in gastric cancer tissue and cells, resulting in tumor metastasis via activation of the PAX3–MET pathway [44,45]. In addition, Azuma et al. showed that exosomal *miR-658* is increased in gefitinib-resistant human lung adenocarcinoma PS-9/ZD cells and is associated with acquisition of chemoresistance in chemosensitive PS-9 cells [46]. Thus, exosome-mediated cancer proliferation and drug resistance requires further investigation for these two miRNAs. Inversely, *miR-381-3p*, *miR4698*, *miR491-3p*, and *miR-2392* are reported to have tumor-suppressive effects. Downregulation of *miR-381-3p* is associated with osteosarcoma growth [47], while *miR-4698* has inhibitory effects on cancer cell proliferation in glioblastoma [48], hepatocellular carcinoma [49], and gastric carcinoma cells [50]. *miR-491-3p* acts as tumor suppressor and metastasis inhibitor in non-small cell lung cancer [51] and gastric cancer [52]. *miR-2392* also has suppressive effects on cancer cell proliferation and metastasis in triple-negative breast cancer [53], hepatocellular carcinoma [54,55], and gastric cancer [56]. Therefore, these candidates may be upregulated in HL-60/ADR exosomes as negative feedback for chemoresistance. However, because the packaging mechanism of miRNA into exosomes via ceramides is largely unknown, this is a topic for future investigation.

## 4. Materials and Methods

### 4.1. Materials

Doxorubicin was obtained from Sigma–Aldrich (St. Louis, MO, USA). Anti-CD81 (sc-7637) and anti-actin (sc-1616) antibodies were purchased from Santa Cruz Biotechnologies (Dallas, TX, USA). Anti-calnexin (610523) antibody was obtained from BD Biosciences (San Jose, CA, USA). Anti-capase-3 antibody (9665S) was from Cell Signaling (Danvers, MA, USA). Horseradish peroxidase-conjugated secondary antibodies were purchased from Promega (Madison, WI, USA). The pLKO.1-TRC cloning vector (Addgene plasmid #10878) and the pLKO.1-TRC control (Addgene plasmid #10879) were gifted from David Root and were originally obtained from Addgene (Cambridge, MA, USA) [57].

### 4.2. Cell Culture

Human leukemia HL-60 cells and HL-60/ADR cells were cultured in RPMI 1640 (Wako, Tokyo, Japan) containing 10% (*v*/*v*) fetal bovine serum (FBS), as described previously [4,58]. HL-60 cells expressing SMS1 or SMS2 were established by the retroviral infection of pDON-hSMS1 (HL-60/SMS1), pDON-hSMS2 (HL-60/SMS2), and empty pDON-AI (HL-60/vec) [59]. Human embryonic kidney fibroblast HEK293 cells were maintained in DMEM supplemented with 10% (*v*/*v*) FBS. *miR-484* knockdown in HEK293 cells was performed by transfection of pLKO.1 vector containing the shRNA sequence for *miR-484* (5′-AAACCCCTAAATAG) with Lipofectamine 3000 (Thermo Fisher Scientific, Rockford, IL, USA). Viable cell numbers were measured by Cell Counting Kit 8 (Wako, Tokyo, Japan) or counted after staining with 0.25% (*w*/*v*) trypan blue.

### 4.3. Exosome Preparation

Exosomes were prepared from the culture media of HL-60 and HL-60/ADR cells as previously described [15]. In brief, cells were cultured in RPMI-1640 containing 5 μg/mL insulin and 5 μg/mL transferrin to avoid induction of cell death. After 24 h, the culture media were collected, centrifuged at 2000× *g* for 10 min, and passed through a 0.45-μm polyvinylidene difluoride (PVDF) filter. Then, media were centrifuged twice at 100,000× *g* for 70 min to obtain the exosomes as pellets. Exosome pellets were resolved in PBS(−) and used for other experiments.

### 4.4. Transmission Electron Microscopy

Transmission electron microscopy was performed as described previously [60]. Briefly, extracted exosomes were put onto carbon-coated copper grids (400 mesh) for 10 s. After washing with PBS, the grids were negatively stained with 2% (*w*/*v*) uranyl acetate for 1 min. Transmission images were obtained using a H-7100 transmission electron microscope (Hitachi, Tokyo, Japan) at 100 kV.

### 4.5. Analysis of Particle Number and Size

The number and size of the extracted exosomes were measured by nanoparticle tracking analysis (NTA) using a NanoSight NS300 (Malvern Instruments Ltd., Malvern, UK) according to the manufacturer’s protocol. For NTA of exosomes, samples were diluted with PBS(−). Three videos of 60 s each were captured with 1498 frames and a camera level at 15 using a syringe pump with constant flow injection. The recorded videos were analyzed with NTA software version 3.2 (Malvern Instruments Ltd., Malvern, UK) to determine the concentration and particle size.

### 4.6. Western Blot Analysis

Cells and exosomes were lysed in RIPA buffer (Wako, Tokyo, Japan) for 20 min on ice. Cell lysates were separated by SDS-polyacrylamide gel electrophoresis and transferred to PVDF membranes (Millipore, Bedford, MA, USA). After incubation with Blocking One solution (Nacalai Tesque, Kyoto, Japan) for 15 min at room temperature, the membranes were incubated overnight at 4 °C with primary antibodies. The membranes were then incubated with horseradish peroxidase-conjugated secondary antibodies for 45 min at room temperature. Immunoreactive protein bands were detected with the LAS-4000 system (Fujifilm, Tokyo, Japan).

### 4.7. LC-MS/MS

Lipid extractions from cells and exosomes were performed as described previously [61]. In brief, cells and exosomes were sonicated for 30 s with 0.1 mL of methanol/butanol (1:1), and then 0.7 mL butanol, 0.05 mL of 0.5 M phosphate buffer (pH 6.0), and 0.2 mL water were added. The samples were shaken, sonicated for 3 min, and centrifugated at 9000× *g* for 5 min. After collection of upper phase, remained solution was re-suspended with 0.35 mL each of hexane and ethyl acetate. After centrifugation at 9000× *g* for 5 min, upper phase was collected and mixed with first upper phase and 0.7 mL of methanol. Ten percent of this solution was dried and used for measurements of sphingolipids including ceramides, SM, GC, and PC with LC-MS/MS (Ultimate 3000 LC system, Thermo Fisher Scientific, Rockford, IL, USA), as described previously [61,62]. The protein concentrations in the residue of lipid extraction were measured by a BCA Protein Assay Kit (Thermo Fisher Scientific, Rockford, IL, USA) and used for normalization.

### 4.8. Measurement of SMS and GCS Activity

The activity of SMS and GCS was assessed using previously described methods [59,63]. Cells were homogenized in buffer containing 20 mM Tris-HCl (pH 7.5), 2 mM EDTA, 10 mM EGTA, and inhibitor cocktail (Roche, Basel, Switzerland). Homogenates (100 μg protein) were incubated in reaction solution (10 mM Tris-HCl (pH 7.5), 1 mM EDTA, 20 μM C_6_-NBD-ceramide (Matreya, Pleasant Gap, PA, USA), 0.1 mM UDP-glucose, 120 μM phosphatidylcholine) and incubated for 60 min at 37 °C. Lipids were extracted using the Bligh–Dyer method [64] applied onto thin layer chromatography plates, and developed with a solvent consisting of chloroform–methanol–12 mM MgCl_2_ (65:25:4, *v/v/v*). The fluorescent lipids were detected using LAS-4000 and quantified using Multi Gauge 3.1 (Fujifilm, Tokyo, Japan).

### 4.9. Quantitative Real-Time PCR (qPCR)

Total RNA and miRNA were extracted using an RNeasy mini kit (Qiagen, Hilden, Germany) and a miRNeasy mini kit (Qiagen, Hilden, Germany), respectively. Total RNA was converted to cDNA using ReverTra Ace qPCR RT Kit (Toyobo, Osaka, Japan). qPCR for *SGMS1* (SMS1; Hs00983630_m1) and *SGMS2* (SMS2; Hs00380453_m1) was performed using the QuantStudio 12K Flex Real-Time PCR system (Thermo Fisher Scientific, Rockford, IL, USA) with TaqMan Probes (Thermo Fisher Scientific, Rockford, IL, USA), as described previously [65]. *SGMS1* and *SGMS2* expression was normalized against *ACTB* (Hs01060665_g1) expression. A TaqMan Advanced miRNA cDNA Synthesis Kit (Thermo Fisher Scientific, Rockford, IL, USA) and TaqMan Advanced miRNA Assay (Thermo Fisher Scientific, Rockford, IL, USA) were used to assess *miR-484* expression (has-miR-484, 478308_mir). In exosomes, 10 ng exosomal miRNA was used for assays and normalized against miRNA concentration. In cells, *miR-484* expression was normalized against *ACTB* expression.

### 4.10. Flow Cytometry

Measurement of apoptotic cells was performed by detection of phosphatidylserine exposure using fluorescein isothiocyanate (FITC)-conjugated Annexin V (BD Biosciences, San Jose, CA, USA) and flow cytometry analysis (Gallios, Beckman Coulter, Miami, FL, USA), as described previously [66]. The percentage of FITC-Annexin V-positive cells was calculated as apoptotic cells using Kaluza software (Beckman Coulter, Miami, FL, USA). Intracellular fluorescence of Dox was detected by flow cytometry equipped with 488 nm excitation and 695 nm emission [67]. Fluorescent intensity was quantified with Kaluza software (Beckman Coulter, Miami, FL, USA) and represented as the mean fluorescent intensity (MFI).

### 4.11. Microarray for miRNA

Total RNA was extracted from exosomes and cells using 3D-Gene RNA extraction reagent (Toray Industries, Inc., Tokyo, Japan). The miRNA microarray analysis was performed with a 3D-Gene miRNA Labeling Kit and a 3D-Gene Human miRNA Oligo Chip version 21 (Toray Industries Inc., Tokyo, Japan) to detect 2565 human miRNAs. The chip was scanned with a 3D-Gene Scanner (Toray Industries Inc., Tokyo, Japan) and analyzed with a 3D-Gene Extraction Software (Toray Industries Inc., Tokyo, Japan). The digitalized fluorescence signal provided by this software was used as raw data. All normalized data were globally normalized per microarray such that the median signal intensity was adjusted to 25. From these data, miRNAs that were expressed in HL-60/ADR exosomes at greater than 5-fold higher levels than in HL-60 exosomes were listed.

### 4.12. Statistical Analysis

Statistical analyses were performed using GraphPad Prism 9 software (GraphPad Software Inc., San Diego, CA, USA). Data are represented as the mean ± standard deviation. Statistical significance was determined using paired *t* tests (between groups) or one-way ANOVA with a follow-up Tukey’s test (between multiple groups). A *p* value of less than 0.05 was considered statistically significant.

## 5. Conclusions

Chemoresistance against anti-cancer drugs is largely a clinical problem in various cancer types. However, the molecular mechanisms involved in drug resistance are diverse, and their elucidation is extremely difficult. In this study, we observed increased exosome numbers in chemoresistant HL-60/ADR cells compared with those in HL-60 cells, leading to acquisition of drug resistance and enhanced proliferation through the exosomal transport of miRNAs including *miR-484*. Promotion of exosome production and release is associated with intracellular ceramide metabolism by SMS2. Exosomes are well-known to be related in the cancer malignancy through transport of their cargo such miRNAs [7,8]. In addition, ceramide generation is also important to exosome production and release [12,13]. Furthermore, it is well known that ceramides are the anti-cancer sphingolipids to induce apoptosis or growth arrest and are kept at low levels in malignant cancer cells [4]. However, their relevance had been unknown. In this study, our results suggest that the balance between SM hydrolysis-mediated ceramide production and steady-state intracellular SM and ceramide levels is associated with ceramide-mediated exosome production and release through intracellular trafficking, results in cancer malignancy including cancer cell proliferation and chemoresistance. Therefore, suppression of exosome release by targeting ceramide metabolism such as inhibition of SMS2 may be a therapeutic strategy for cancers such as leukemia.

## Data Availability

Not applicable.

## Supplementary Materials

The following supporting information can be downloaded at: https://www.mdpi.com/article/10.3390/ijms231810648/s1.
