# Supplementary material for: Ceramide Metabolism Regulated by Sphingomyelin Synthase 2 Is Associated with Acquisition of Chemoresistance via Exosomes in Human Leukemia Cells
Source: Int J Mol Sci. 2022 Sep 13;23(18):10648. doi: 10.3390/ijms231810648 (PMC9505618; doi:10.3390/ijms231810648)

Supplemental data

Ceramide metabolism regulated by sphingomyelin synthase 2 is associated with acquisition of chemoresistance via exosomes in human leukemia cells

Makoto Taniguchi, Shingo Nagaya, Kohei Yuyama, Ai Kotani, Yasuyuki Igarashi, and Toshiro Okazaki.

Figure Legends

Figure S1 Full-length blots of Figure 1B.

Figure S2 Glucosylceramide synthase (GCS) activity and measurement of hexocylceramide (HexCer). (A) Sphingomyelin synthase (SMS) and GCS activities were measured by using fluorescent C_6-NBD-ceramide and thin layer chromatography method described in “Materials and Methods.” Data of SMS activity was in Figure 3A. (B) HexCer levels were measured by LC-MS/MS. The values show the mean ± SD (n = 3). **P* < 0.05, ***P* < 0.005.

Figure S3 HL-60/ADR cells exhibited doxorubicin (Dox) resistance. Cells were treated with 0.25 μM of Dox for 12 hours. (A) Viable cells were counted after trypan blue exclusion and indicated as percent of vehicle treatment (0 μM) (n = 4). (B) Apoptotic cells were detected by FITC-annexin V staining and

flowcytometry (n = 3). (C) Western blot analysis of caspase-3 and actin. (D) Dox elimination was measured by flowcytometry (n = 3). The values show the mean ± SD.

Figure S4 HL-60 exosome did not confer chemoresistance on HL-60 cells. HL-60 cells were pre-treated with HL-60 exosomes (HL-60 exo) for 12 h and then treated with doxorubicin (Dox) for 12h. Viable cells were counted after trypan blue exclusion and indicated as percent of vehicle treatment (n = 3). The values show the mean ± SD. $^{**}P < 0.005$.

Supplemental Figure S1

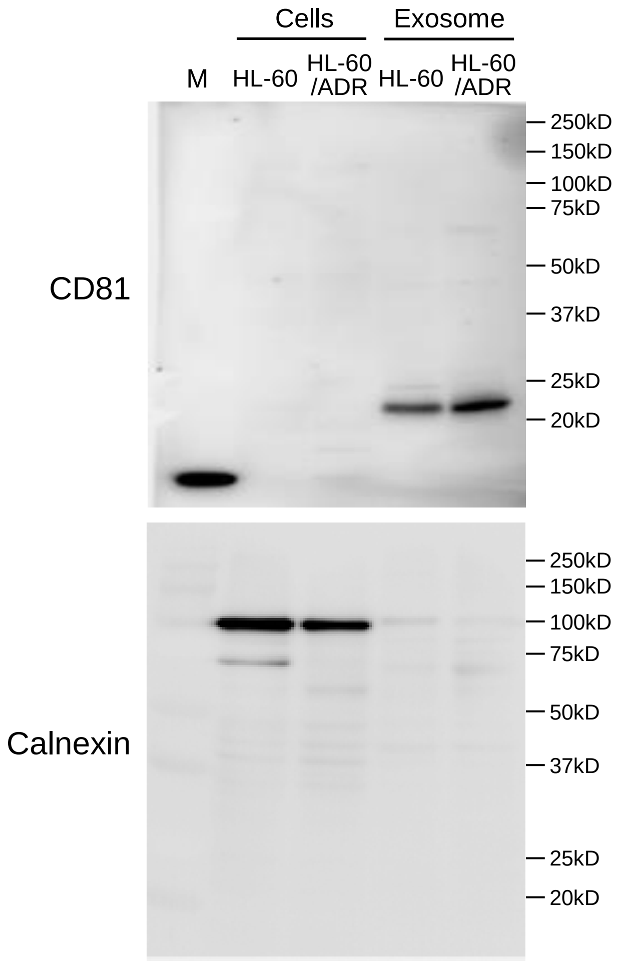

Supplemental Figure S2

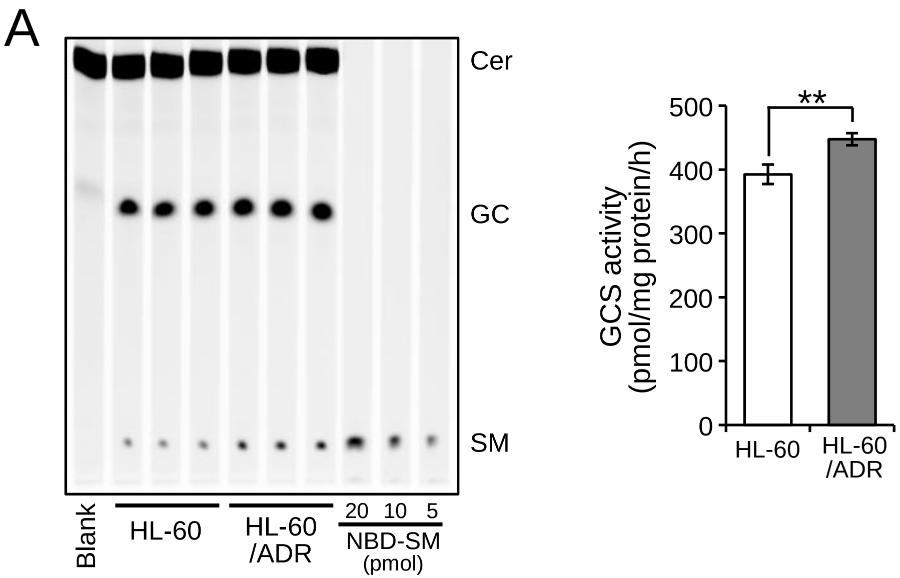

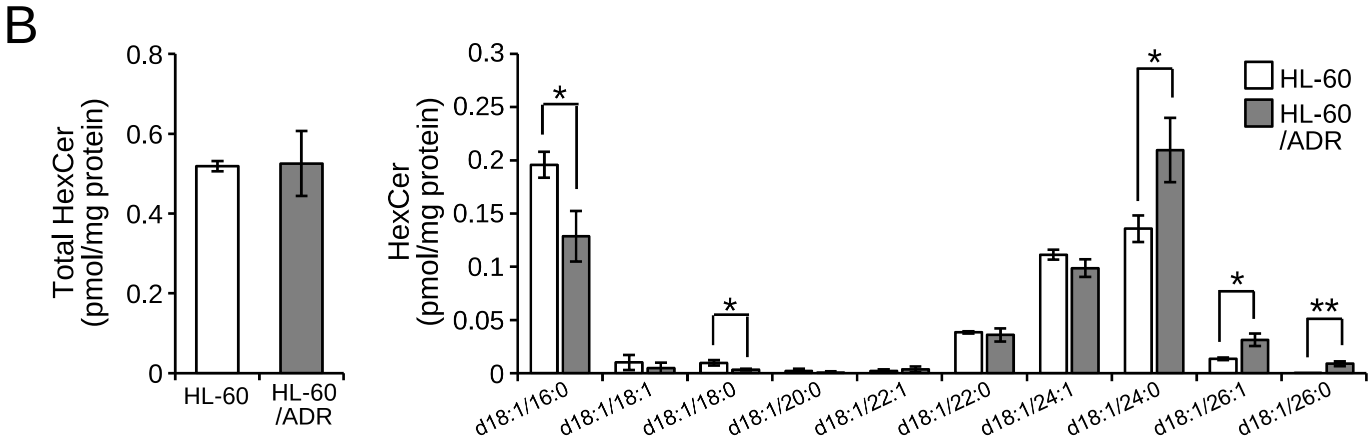

Supplemental Figure S3

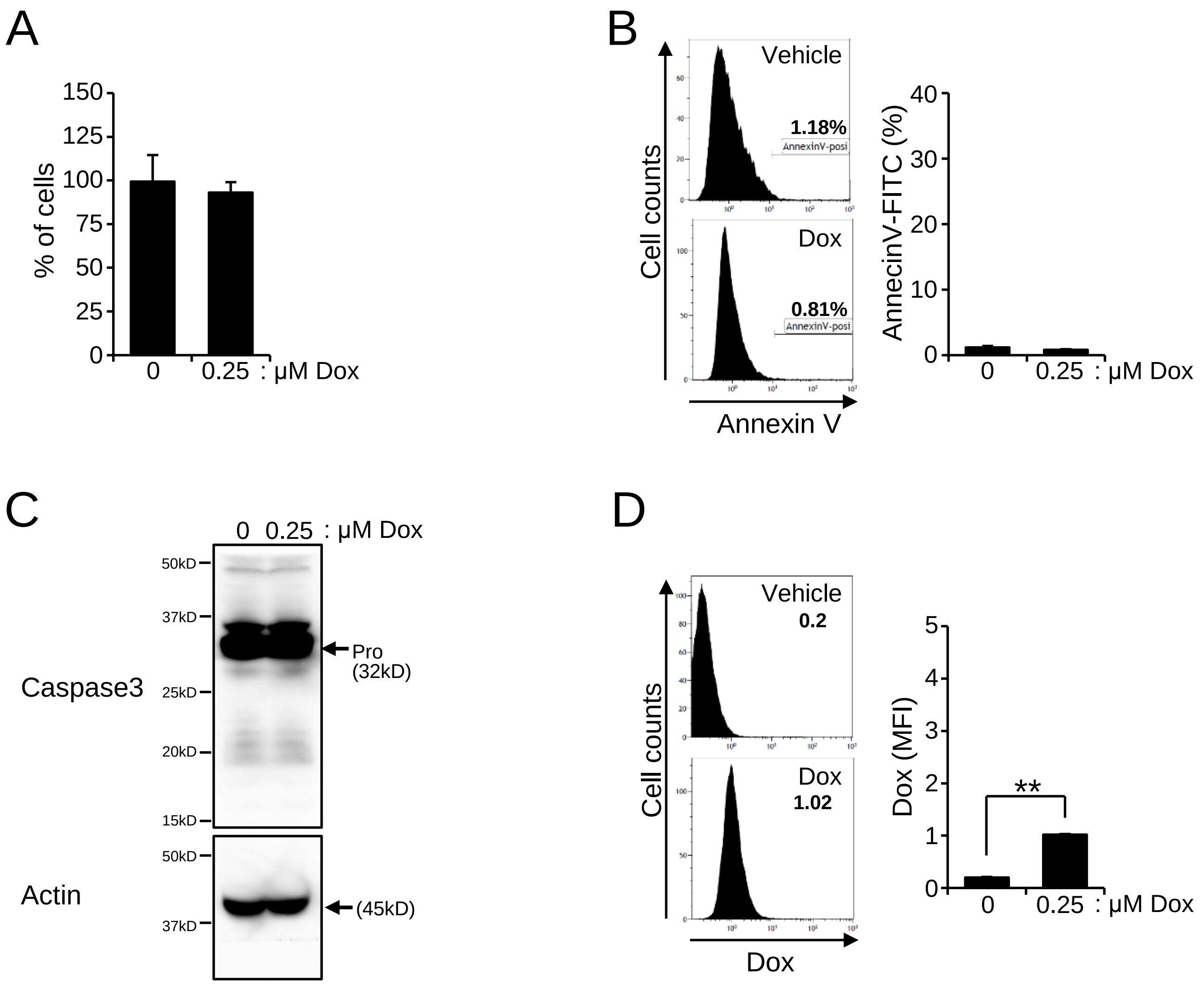

Supplemental Figure S4

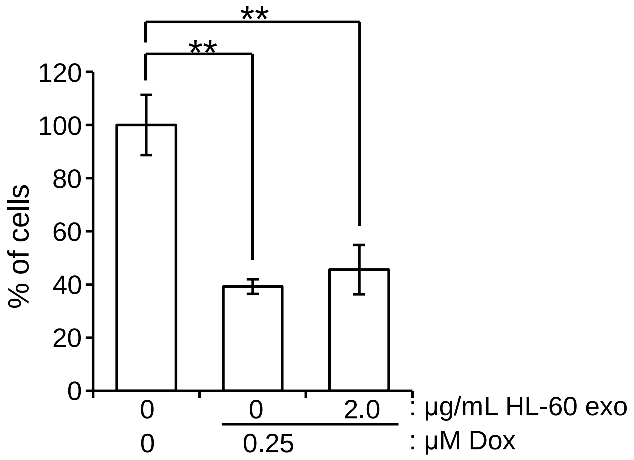

Supplement: Supplementary file 1 [file ijms-23-10648-s001.zip › ijms-1893554-supplementary.pdf]
